# Thioester derivatives of the natural product psammaplin A as potent histone deacetylase inhibitors

**DOI:** 10.3762/bjoc.9.11

**Published:** 2013-01-15

**Authors:** Matthias G J Baud, Thomas Leiser, Vanessa Petrucci, Mekala Gunaratnam, Stephen Neidle, Franz-Josef Meyer-Almes, Matthew J Fuchter

**Affiliations:** 1 Department of Chemistry, Imperial College London, London SW7 2AZ, United Kingdom; 2 Department of Chemical Engineering and Biotechnology, University of Applied Sciences, Schnittspahnstraβe 12, 64287 Darmstadt, Germany; 3 Cancer Research UK Biomolecular Structure Group, UCL School of Pharmacy, 29-39 Brunswick Square, London WC1N 1AX, United Kingdom

**Keywords:** epigenetics, histone deacetylase, natural product, prodrug, psammaplin A, thioester

## Abstract

There has been significant interest in the bioactivity of the natural product psammaplin A, most recently as a potent and isoform selective HDAC inhibitor. Here we report our preliminary studies on thioester HDAC inhibitors derived from the active monomeric (thiol) form of psammaplin A, as a means to improve compound delivery into cells. We have discovered that such compounds exhibit both potent cytotoxicity and enzymatic inhibitory activity against recombinant HDAC1. The latter effect is surprising since previous SAR suggested that modification of the thiol functionality should detrimentally affect HDAC potency. We therefore also report our preliminary studies on the mechanism of action of this observed effect.

## Introduction

Chromatin is a macromolecular complex consisting of DNA, histone and nonhistone proteins. The epigenetic control of chromatin organization plays a major role in the regulation of gene expression, and consequently cell differentiation, proliferation and survival. Such control is mediated by a myriad of remodelling proteins, able to bind to, and covalently modify, chromatin [1–2]. In recent years, it has become increasingly apparent that misregulation of epigenetic pathways contributes to oncogenesis [3–4] and small molecule inhibitors of these pathways have emerged as highly attractive targets for anticancer therapies [5–6]. Inhibitors of epigenetic pathways should not only be useful as anticancer drugs, but also as molecular probes to study the causative relationships between specific epigenetic modifications, their biological outcomes, and how their misregulation is involved in diseases such as cancer [1–2].

The dynamic post-translational acetylation/deacetylation of histone proteins is one of the most commonly studied epigenetic events, and occurs at specific lysine residues on the N-terminal histone tails, which project out from the nucleosome (the fundamental repeating unit of chromatin). Acetylation/deacetylation of such lysine residues is achieved by the action of histone acetyltransferases (HATs) and histone deacetylases (HDACs), respectively. Histone deacetylation by HDACs causes transcriptional repression through both chromatin condensation and chromatin signalling. To date, 18 human genes encoding proven or putative HDACs have been identified [7]. HDACs fall into two categories: the zinc-dependent enzymes (class I, II and IV) and the NAD^+^-dependent enzymes (class III, also called sirtuins) [5]. Class I HDACs (HDAC1, 2, 3, 8) are mostly present in the nucleus, whereas class II HDACs are tissue specific and shuttle between the cytoplasm and the nucleus [8–9]. Class II can be further subdivided into class IIa (HDAC4, 5, 7, 9) and class IIb (HDAC6, 10). HDAC11 constitutes its own class IV. Despite their name, several HDACs are able to deacetylate a number of nonhistone protein substrates [10–11]. Sirtuins are structurally and mechanistically distinct enzymes.

**Figure 1:**
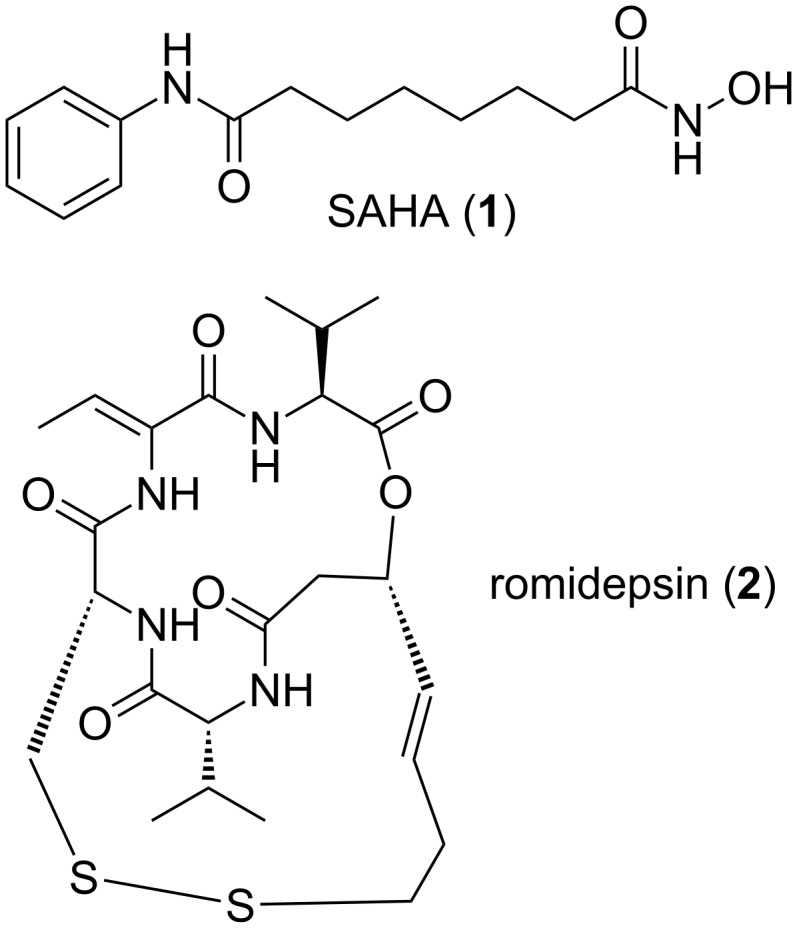
FDA approved HDAC inhibitors for the treatment of CTCL.

To date, only two compounds that inhibit HDACs have been FDA approved: suberoylanilide hydroxamic acid (SAHA, **1,** trade name Zolinza by Merck & Co.) and romidepsin **2** (trade name Istodax by Celgene) for the treatment of cutaneous T-cell lymphoma (CTCL, Figure 1) [12–14]. The success of these compounds in the clinic has led to a significant interest in the further discovery of structurally novel HDAC inhibitors that, in particular, exhibit improved isoform selectivity.

**Scheme 1:**
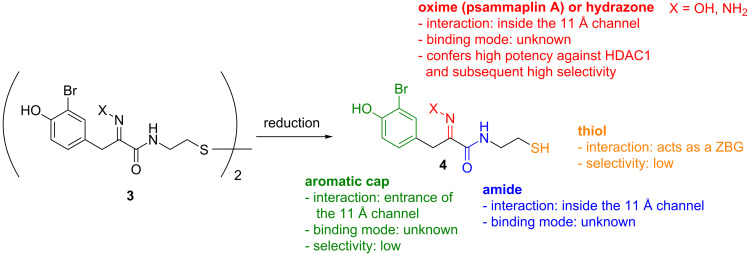
SAR of psammaplin A against zinc-dependant HDACs. Adapted from Baud et al. [20].

Among the myriad of previously reported HDAC inhibitors, psammaplin A [15–18] (**3**, X = OH, Scheme 1, left) displays an intriguing structure. It is a symmetric, dimeric hydroxyiminotyrosine-based natural product, characterised in 1987, and represents the first example of a disulfide and oxime containing metabolite isolated from a marine sponge. Since its initial report by Crews and co-workers as a potent HDAC inhibitor [16], psammaplin A has provided inspiration for the development of new HDAC inhibitors with novel structures [19]. Recently, we [20] and others [21] reported an in-depth structure–activity relationship of this natural product against its HDAC targets. Dissection of its activity against a panel of HDACs allowed us to highlight structural features responsible for its high inhibitory potency and selectivity. In particular, we unambiguously demonstrated that, similarly to the natural product and clinically approved romidepsin, psammaplin A is a prodrug, requiring reduction of its disulfide functionality to the corresponding thiol monomer **4** (X = OH), in order to potently inhibit HDACs (Scheme 1, right). The resulting thiol moiety acts as a zinc binding group within the active site of the HDAC protein. Furthermore, we demonstrated the importance of the oxime unit of psammaplin A and related analogues for high potency and selectivity against recombinant HDAC1 (rHDAC1) in vitro (Scheme 1). More recently, we disclosed highly potent heterocyclic *N*-thioethylamide-based HDAC inhibitors based on the psammaplin A pharmacophore and rationalised the results using computational modelling [22].

While prereduced psammaplin A and thiol-containing analogues displayed nanomolar to subnanomolar potencies in vitro, they only displayed modest potencies in cell-based assays against A549 (human lung carcinoma), MCF7 (human breast carcinoma) and WI38 (normal human lung fibroblast) cell lines. We attributed this to the low permeability and/or stability of the free thiol in cells. While the use of nonreduced disulfide functionality (e.g., present in the parental psammaplin A (**3**), X = OH) is one strategy to “protect” the free thiol and allow for its effective dosing into cells, this prodrug strategy is reliant on intracellular reduction of the disulfide to the active thiol form. As such, cellular potency would be expected to correlate significantly with the cellular levels of reductants such as glutathione [23]. An alternative prodrug approach to “protect” the thiol active form of psammaplin A analogues would be to form the corresponding thioester; the active thiol being generated in cells after cleavage of the acyl group by nonselective esterases. In support of this approach, Miyata and co-workers synthesised a number of SAHA-derived thioesters during their research of nonhydroxamate inhibitors of HDACs, which exhibited moderate to high potency in enzymatic and cell-based assays [24–25]. Interestingly, the potencies of their compounds were higher than the potencies of their corresponding dimeric disulfide analogues, and this was thought to reflect the rate of thioester hydrolysis versus the rate of disulfide reduction. We therefore commenced a study to prepare thioester derivatives of our psammaplin A analogues to investigate whether this would be an effective strategy to optimise these potent and selective HDAC inhibitors.

## Results and Discussion

### Synthesis of acetate-protected thiol analogues of psammaplin A

**Scheme 2:**
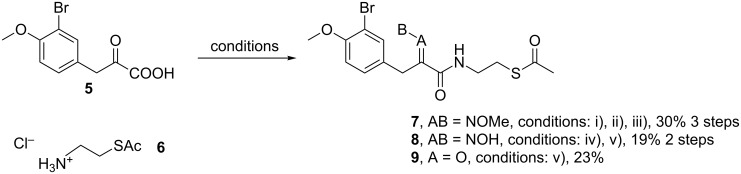
Synthesis of **7–9**. Conditions: (i) HCl·H_2_NOMe, pyridine, rt, 12 h; (ii) EDC, NHS, dioxane, rt, 3 h; (iii) **6**, Et_3_N, dioxane/MeOH, rt; (iv) HCl·H_2_NOH, pyridine, rt, 12 h; (v) DCC, NOHP, **6**, Et_3_N, dioxane, rt, 24 h.

We designed and synthesised several acetate-protected psammaplin A analogues. The structures of our thioester-based probes are shown in Scheme 2. We recently demonstrated [20] that variation of the aromatic substitution pattern had only a marginal influence on the HDAC inhibitory potency of psammaplin A analogues in vitro. Therefore, the native phenol was replaced by its methylated homologue for ease of synthesis, notably to avoid side reactions during the carbodiimide-mediated coupling step, whereby we had previously found the phenol to act as a competitive nucleophile. Since we had previously demonstrated the importance of the oxime (Scheme 1) for HDAC potency and selectivity, we prepared probes with these different functionalities in order to allow comparison of our data to our previously generated in vitro and in cell SAR data [20].

Condensation between acid **5** and *O*-methylhydroxylamine, followed by EDC coupling with *S*-2-aminoethyl ethanethioate **6** [26] afforded thioacetate analogue **7** (Scheme 2). Condensation of acid **5** with hydroxylamine, followed by coupling with **6** using DCC and *N*-hydroxyphthalimide [27] as coupling reagents afforded thioacetate **8**. The latter conditions were also applied to **5** and afforded thioacetate analogue **9**. The isolated yields for products **8** and **9** were unoptimised, and our previous work suggests that these could be improved with further refinement of the reaction conditions [28].

Thioesters **7**–**9** were assayed against A549, MCF7 and WI38 cell lines, in addition to recombinant human rHDAC1 (class I) and recombinant human HDAC6 (rHDAC6, class II) as previously reported [20]. Psammaplin A (**3**), prereduced psammaplin A thiol (**4**), and SAHA (**1**) were included as control compounds. The results are shown in Table 1. IC_50_^6/1^ is defined by the ratio IC_50_^HDAC6^/IC_50_^HDAC1^ and was used as an indicator of isoform selectivity in vitro.

**Table 1:** Biological data.

Compound	A549 IC_50_ (μM)	MCF7 IC_50_ (μM)	WI38 IC_50_ (μM)	rHDAC1 IC_50_ (μM)	rHDAC6 IC_50_ (μM)	IC_50_^6/1^

SAHA (**1**)	n.d.	n.d.	n.d.	0.030	0.21	7
PSA (**3**)	7.5	1.3	3.4	0.045	2.8	62
PSA-SH (**4**)	2.5	2.4	3.4	0.001	0.36	360
**8**^a^	8.3 (4.1/0.16)	3.2 (0.63/0.61)	5.2 (2.2/1.1)	0.005 (0.043/0.002)	23 (2.0/3.2)	4560 (46/1772)
**7**^a^	44 (46/5.1)	12 (13/12)	17 (n.d./10)	0.12 (0.22/0.015)	>50 (>50/2.7)	>417 (>230/180)
**9**^a^	>50 (10/11)	21 (3.9/3.4)	>50 (4.1/14)	0.48 (9.0/1.1)	9.5 (8/0.25)	>20 (0.9/0.22)

^a^In brackets are data for the corresponding disulfides and thiols respectively, obtained from Baud et al [20]*.*

The synthesised thioesters displayed modest but significant cytotoxic activity in our cell-based assays (Table 1, columns 1–3). Compound **8**, which contains an oxime moiety, was the most potent compound in each case, followed by methyloxime **7**, and finally ketone **9**. These findings parallel our previous SAR data for the corresponding thiols [20], and reflect the previously established potency ranking for the AB system: NOH > NOMe > O. The most sensitive cell line to treatment was the MCF7 breast cancer cell line, with the most potent thioester **8** displaying an IC_50_ of 3.2 μM. Such sensitivity correlates with previous SAR data [20]. While being moderately potent against MCF7 cells (21 μM), **9** was inactive against A549 and WI38 cells at the highest concentration tested (50 μM). Similar to our previously reported SAR and mechanistic studies, a correlation between HDAC inhibition and cytotoxicity is clearly observable for these psammaplin A prodrugs.

Notably, in enzyme assays, the potencies of our acetate-protected analogues approached those of their corresponding thiols (Table 1, see note^a^), while being approximately 10-fold more potent than the parental disulfide series (Table 1, see note^a^). For example, **8** was found to be highly potent against rHDAC1, displaying an IC_50_ of 5 nM. With the exception of **9**, acetyl-protected compounds were found to be 2.5 (**8**) to 8 times (**7**) less potent than the corresponding free thiol analogues against rHDAC1. Curiously, acetyl-protected compounds **7**–**9** were found to be moderately active to completely inactive against rHDAC6 in each case, highlighting the important isoform selectivity of these compounds.

The mechanistic origin for the high potency of the supposed prodrug thioesters in cell-free assays was unclear: Such compounds were designed to be cleaved to give the active (thiol) inhibitor in cells, and presumably exhibit a decreased potency against the target HDAC, prior to cleavage of the acetyl group. As previously mentioned, Miyata and co-workers synthesised a number of SAHA-based thioesters, which exhibited moderate to high potency in both enzymatic and cell based assays. The enzymatic assays they employed, however, used cell extracts as the source of HDACs, potentially containing esterases, which may have cleaved the thioester during the assay. On the contrary, we observed the same pattern with purified rHDAC1 and purified rHDAC6, which obviously contains no such potential for esterase-driven hydrolysis. Recently, Williams reported the high potency of the natural product largazole, bearing an octanoyl-protected thiol, against HDACs [29]. While the deprotected analogue displayed nanomolar to subnanomolar potency in vitro against purified HDACs, the native thioester was still highly potent. They hypothesized that the octanoyl group was cleaved in situ to liberate the active thiol; however, no rigorous studies were reported to confirm this.

**Scheme 3:**
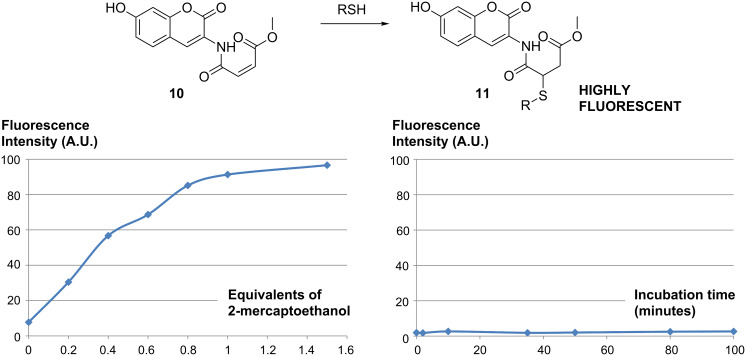
Top: Generation of the fluorescent adduct **11** after reaction of probe **10** with thiols. Bottom left: Fluorescence intensity at 465 nm (Y-axis, A.U.) as a function of the number of equivalents of 2-mercaptoethanol added (X-axis). Bottom right: Fluorescence intensity at 465 nm (Y-axis, A.U.) as a function of the incubation time (X-axis, minutes) of probe **10** with thioester **8**.

We therefore undertook preliminary studies to attempt to shed light on the reasons for the high potency of our thioesters in the enzymatic assays. We first envisaged that our assay buffer could potentially be responsible for thioester hydrolysis. We prepared and used synthetic probe **10** [30] (Scheme 3) in order to test this hypothesis. Coumarin-based probe **10** is known to react extremely fast with thiols through a Michael addition reaction. While the fluorescence of **10** is efficiently quenched by the intramolecular double bond, upon reaction with thiols, a highly fluorescent conjugate **11** is produced. This system has been found to be particularly efficient to quantify thiol concentration in biological systems [30].

Control experiments were performed using 2-mercaptoethanol as the thiol source [30]. Fluorescence spectra of **10** (10^−6^ M **10** in buffer FB-188 [31]) were recorded after incubation with 0, 0.2, 0.4, 0.6, 0.8, 1.0 and 1.5 equiv 2-mercaptoethanol (Scheme 3, bottom left). As expected, a linear relationship between fluorescence intensity and the concentration of thiol could be observed between 0 and 1.0 equiv, confirming the sensitivity and reliability of this system at low concentration. When our HDACi thioester **8** was incubated with probe **10** (Scheme 3, bottom right) under the buffer conditions, however, no significant variation of the fluorescence intensity was observed. The standard incubation time of thioesters in our HDAC inhibitor assay is 20–30 minutes; however, no evidence of thiol formation was observed after up to 100 minutes of incubation in the assay buffer. This data therefore does not support the hydrolysis of our thioester inhibitors in the assay buffer. Two explanations could be envisaged to explain this result: (1) The thioester is stable to the assay buffer and therefore does not contribute to the in situ generation of the free thiol. (2) The quantity of free thiol generated in situ in this experiment was too low to be quantified.

**Figure 2:**
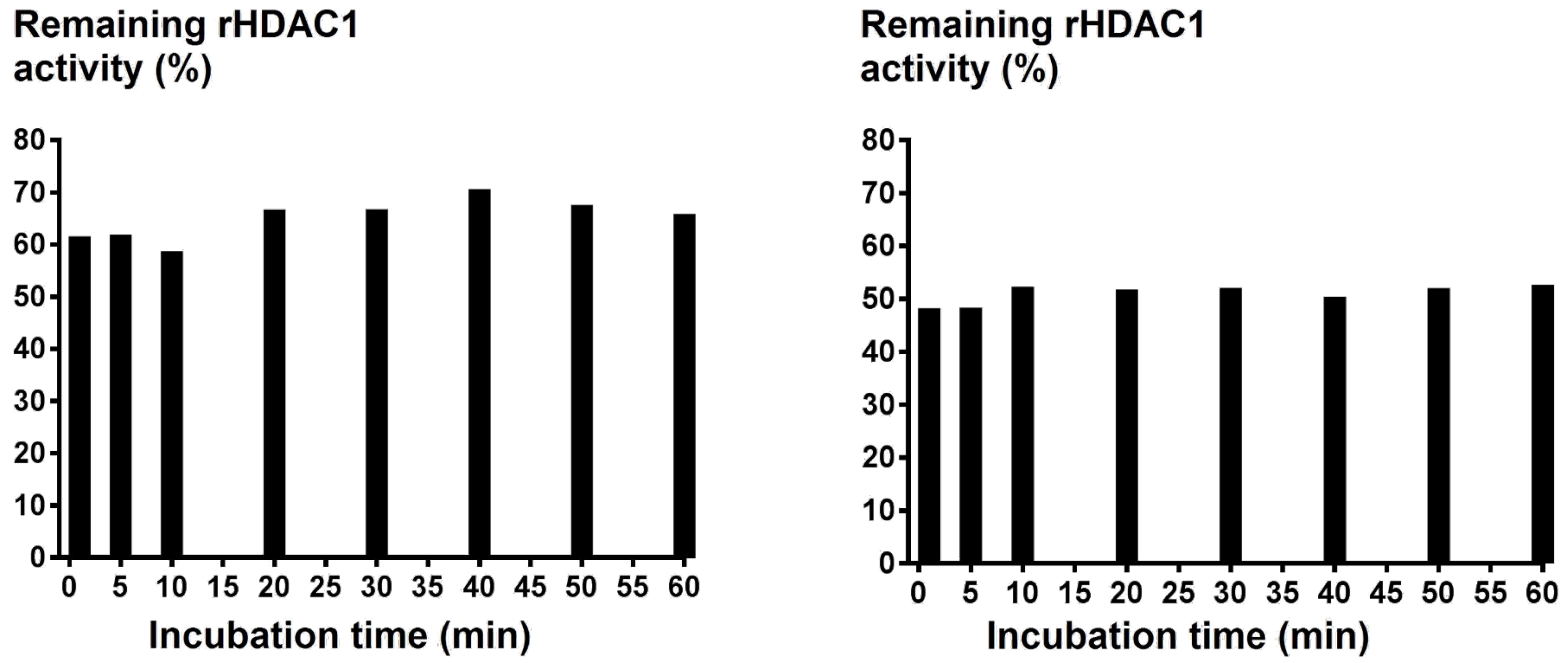
rHDAC1 was incubated with a predetermined IC_50_ concentration of **7** (left) and **9** (right) for 1–60 minutes, and the remaining rHDAC1 activity (Y-axis, %) was recorded and plotted as a function of the incubation time (X-axis, minutes).

An alternative hypothesis for the high potency of the thioester inhibitors is that the HDAC enzyme cleaves the acetyl group directly, utilizing its intrinsic deacetylase activity. Inhibition of rHDAC1 was measured (at the IC_50_ concentration of thioester) as a function of the initial incubation time (1–60 minutes) of rHDAC1 with thioesters **7** and **9** (Figure 2). No variation of enzymatic inhibition could be observed in each case, excluding rHDAC1 as the source of hydrolysis. Finally, we assessed whether the use of thioester inhibitors could influence the assay readout, when using our coupled HDAC assay. Trypsin-dependant generation of the fluorescence intensity (2^nd^ step of our coupled assay) was discounted as a source of false-positive activity, since trypsin was used in a large excess (430 μM) compared to the thioesters (low micromolar to nanomolar). Direct compound inhibition of trypsin would therefore not be significant in the overall readout on stoichiometry grounds.

## Conclusion

In conclusion, we have demonstrated that highly potent and selective HDAC inhibitors can be discovered by preparing thioester derivatives of the natural product psammaplin A and close analogues. Such thioesters display significant cytotoxicity against several cancer cell lines. While initially envisaged as a prodrug approach, we found these thioesters to retain highly potent enzymatic activity using purified HDAC enzymes. Our preliminary results in the investigation of the origin of this effect have discounted hydrolysis of the thioester under the buffered conditions of the assay and direct cleavage of the acetyl group by the deacetylase enzyme. It therefore remains highly plausible that the thioacetate group can function as a potent zinc-binding group in its own right. While this hypothesis requires further validation, it opens up exciting new possibilities to prepare HDAC inhibitors bearing diverse thioester zinc-binding groups. Variation of the thioester functionality to protect its lability in cells and potentially orient suitable functionality into the internal cavity of HDACs [32] remain exciting avenues for future research.

## Experimental

### (*E*)-(*S*)-2-(3-(3-Bromo-4-methoxyphenyl)-2-(methoxyimino)propanamido)ethyl ethanethioate (**7**)


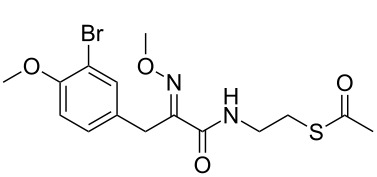


Compound **7** (216 mg, 30% from acid **5**) was prepared according to previously reported procedures [28] and obtained as a yellowish oil after purification by flash column chromatography (AcOEt/CH_2_Cl_2_, 2:98). *R*_f_ 0.35 (AcOEt/CH_2_Cl_2_ 2:98); IR: 1674, 1520, 1495, 1044 cm^−1^; ^1^H NMR (400 MHz, CDCl_3_) δ 2.33 (s, 3H, CH_3_C), 3.04 (t, *J* = 6.6 Hz, 2H, CH_2_S), 3.48 (m, 2H, CH_2_N), 3.81 (s, 2H, 4-CH_2_), 3.84 (s, 3H, CH_3_O-1), 4.01 (s, 3H, CH_3_O-N), 6.78 (d, *J* = 8.4 Hz, 1H, 2-H), 6.97 (br t, 1H, NH), 7.21 (dd, *J* = 8.4, 2.1 Hz, 1H, 3-H), 7.46 (d, *J* = 2.1 Hz, 1H, 5-H); ^13^C NMR (100 MHz, CDCl_3_) δ 28.4, 28.6, 30.6, 39.1, 56.2, 63.1, 111.4, 111.8, 129.4, 129.8, 133.9, 151.4, 154.4, 162.6, 195.6; HRMS (ESI^+^) *m*/*z*: [M + H]^+^ calcd for C_15_H_20_BrN_2_O_4_S, 403.0322; found, 403.0336; Anal. calcd for C_15_H_19_BrN_2_O_4_S: C, 44.67; H, 4.75; N, 6.95; found: C, 44.74; H, 4.78; N, 6.89.

### (*E*)-(*S*)-2-(3-(3-Bromo-4-methoxyphenyl)-2-(hydroxyimino)propanamido)ethyl ethanethioate (**8**)


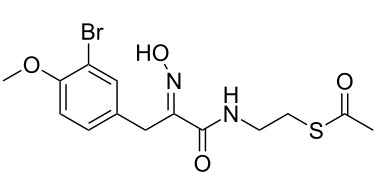


To a solution of crude acid **5** (1 equiv) in freshly distilled pyridine (2 mL/mmol), under argon, was added hydroxylamine hydrochloride (1.5 equiv). The resulting mixture was stirred overnight at rt. Pyridine was then removed in vacuo, and the residue was dissolved in 1 N HCl (5.5 mL/mmol) and extracted three times with ethyl acetate (5.5 mL/mmol). The combined organic layers were dried (Na_2_SO_4_), filtered and concentrated to afford the relatively pure oxime intermediate, used for the next step without further purification. To a solution of this crude oxime derivative in dioxane (10 mL/mmol) under argon was added DCC (1 equiv) and *N*-hydroxyphthalimide (1 equiv). After 2 hours of stirring at rt, 2-acetylsulfanylethylammonium chloride (**6**, 1 equiv) and triethylamine (2.1 equiv) were added, and the resulting mixture was stirred overnight at rt. On the following day, the solvent was evaporated, and the product (50 mg, 19% from acid **5**) was obtained as a white powder after purification by flash column chromatography (AcOEt/CH_2_Cl_2_ 2:8) and trituration in warm AcOEt. Mp 167–169 °C; *R*_f_ 0.6 (AcOEt/CH_2_Cl_2_, 2:8); ^1^H NMR (400 MHz, CD_3_OD) δ 2.29 (s, 3H, CH_3_C), 3.01 (t, *J* = 6.6 Hz, 2H, CH_2_S), 3.41 (m, 2H, CH_2_N), 3.82 (s, 2H, 4-CH_2_), 3.83 (s, 3H, CH_3_O), 6.91 (d, *J* = 8.5 Hz, 1H, 2-H), 7.22 (dd, *J* = 8.5, 2.1 Hz, 1H, 3-H), 7.44 (d, *J* = 2.1 Hz, 1H, 5-H); ^13^C NMR (100 MHz, CDCl_3_) δ 28.7, 29.4, 30.4, 39.9, 56.7, 112.1, 113.1, 130.5, 131.8, 134.7, 152.9, 155.9, 165.9, 197.1; HRMS (ESI^+^) *m*/*z*: [M + H]^+^ calcd for C_14_H_18_BrN_2_O_4_S, 389.0165; found, 389.0164; Anal calcd for C_14_H_17_BrN_2_O_4_S: C, 43.20; H, 4.40; N, 7.20; found: C, 43.29; H, 4.32; N, 7.32.

### (*S*)-2-(3-(3-Bromo-4-methoxyphenyl)-2-oxopropanamido)ethyl ethanethioate (**9**)


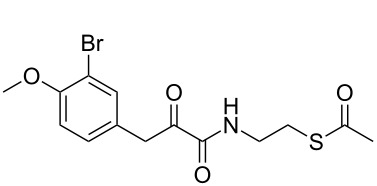


To a solution of acid **5** under argon in dioxane (5.5 mL/mmol) was added DCC (1 equiv), *N*-hydroxyphthalimide (1 equiv). After 2 hours of stirring at rt, 2-acetylsulfanylethylammonium chloride (**6**, 1 equiv) and triethylamine (2.1 equiv) were added and the resulting mixture was stirred overnight at rt. The day after the solvent was evaporated and the product (64 mg, 23%) was purified by flash column chromatography (AcOEt/CH_2_Cl_2_ 1:9) and trituration in Bu_2_O. Mp 93–95 °C; *R*_f_ 0.65 (AcOEt/CH_2_Cl_2_, 1:9); IR: 1685, 1523, 1498, 1106 cm^−1^; ^1^H NMR (400 MHz, CDCl_3_) δ 2.35 (s, 3H, CH_3_C), 3.05 (t, *J* = 6.4 Hz, 2H, CH_2_S), 3.50 (m, 2H, CH_2_N), 3.88 (s, 3H, CH_3_O), 4.13 (s, 2H, CH_2_C(O)), 6.86 (d, *J* = 8.5 Hz, 1H, 2-H), 7.16 (dd, J = 8.5, 2.1 Hz, 1H, 3-H), 7.21 (br t, 1H, NH), 7.42 (d, *J* = 2.1 Hz, 1H, 5-H); ^13^C NMR (100 MHz, CDCl_3_) δ 28.3, 30.6, 39.3, 41.7, 56.2, 111.7, 112.0, 125.9, 130.0, 134.5, 155.1, 159.9, 195.2, 195.5; HRMS (ESI^+^) *m*/*z*: [M + H]^+^ calcd for C_14_H_17_BrNO_4_S, 374.0056; found, 374.0061; Anal calcd for C_14_H_16_BrNO_4_S: C, 44.93; H, 4.31; N, 3.74; found: C, 45.04; H, 4.27; N, 3.81.

### HDAC assays

HDAC assays were performed as previously reported [20]. The recombinant human histone deacetylases rHDAC1 and rHDAC6 were obtained from BPS Bioscience (US).

All reactions were performed in black half area 96-well microplates (Greiner bio-one, Germany) according to the general procedure described by Wegener et al. with some minor modifications. The reaction buffer contained 50 mM KH_2_PO_4_/K_2_HPO_4_, 15 mM Tris/HCl, pH 8, 250 mM NaCl, 0.001% (v/v) Pluronic, and 250 µM EDTA. The buffer components were purchased from Merck (Germany), Roth (Germany) and Sigma-Aldrich.

A serial dilution of test compounds was pre-incubated with 7.4 nM rHDAC1 or 2.8 nM rHDAC6, at 21 ± 1 °C in the dark for different periods of time as indicated. The enzyme reaction was initiated by the addition of Boc-Lys(Ac)-AMC substrate. The reaction mixture was incubated at 30 °C in the dark and stopped after 60 min by the addition of a mixture of 70 µM trypsin and 200 nM SAHA. The fluorescence of AMC served as an indirect measure of HDAC enzyme activity. The kinetics of AMC release was measured on a PolarStar fluorescence plate reader (BMG) with an excitation wavelength of 340 nm and an emission wavelength of 460 nm. Complete cleavage of deacetylated Boc-Lys-AMC by trypsin was achieved after about 10–15 min. The fluorescence intensity of the plateau was averaged over at least 5 min and normalized with respect to the percentage of enzyme activity. Finally, the normalized fluorescence intensities were plotted versus the concentration of test compounds and fitted to a four-parameter logistic model to calculate the IC_50_ values.
